# Photodegradation of Polyethylene Terephthalate and Bis(2-hydroxyethyl) Terephthalate Using Excimer Lamps and Hydrogen Peroxide: A Strategy for PET–Derived Waste Treatment

**DOI:** 10.3390/molecules30153302

**Published:** 2025-08-07

**Authors:** Ángel Navarro-García, María Gómez, María D. Murcia, Elisa Gómez, Asunción M. Hidalgo, Luis A. Dorado, Josefa Bastida

**Affiliations:** Department of Chemical Engineering, Campus de Espinardo, University of Murcia, 30100 Murcia, Spain; a.navarrogarcia@um.es (Á.N.-G.); maria.gomez@um.es (M.G.); md.murcia@um.es (M.D.M.); egomez@um.es (E.G.); ahidalgo@um.es (A.M.H.); langel.dorado@um.es (L.A.D.)

**Keywords:** microplastic, bis(2-hydroxyethyl) terephthalate, terephthalic acid, polyethylene terephthalate, advanced oxidation, excimer radiation

## Abstract

Polyethylene terephthalate (PET) is a widely used polymer whose accumulation in the environment poses a significant pollution challenge. This study explores the degradation of bis(2-hydroxyethyl) terephthalate (BHET) and terephthalic acid (TPA)—two monomers commonly produced during PET hydrolysis and widely used as intermediates in PET recycling—through Advanced Oxidation Processes (AOPs) employing KrCl (222 nm) and XeBr (283 nm) excimer lamps in the presence of hydrogen peroxide (H_2_O_2_). The effects of the H_2_O_2_/monomer mass ratio, initial monomer concentrations, and reaction volume on degradation efficiency were systematically evaluated. The results demonstrate that excimer lamp technology, particularly KrCl, holds promising potential for the effective degradation of both BHET and TPA, and thus represents a viable strategy for PET waste treatment.

## 1. Introduction

Polyethylene terephthalate (PET) is a widely used polymer in various sectors, including packaging, textiles, and consumer goods, owing to its desirable properties, such as versatility, lightweight nature, and durability. However, the rapid expansion of plastic production, reaching hundreds of millions of tons annually, has inadvertently led to severe environmental pollution [1]. A major challenge associated with PET is its inherent persistence; as a non-biodegradable material, it can remain in natural environments for centuries, significantly contributing to vast waste accumulation and ecosystem degradation. This widespread contamination poses substantial risks to both ecosystems and human health. Plastic fragments, including PET, can break down into smaller micro- and nanoplastics [2], which can infiltrate the food chain and potentially induce adverse toxicological and physiological effects on living organisms [3].

Traditional wastewater treatment plants are largely ineffective in removing these persistent organic pollutants, especially at the low concentrations typically found in effluents.

Researchers have investigated several removal methods, such as coagulation, sand filtration, membrane filtration, and adsorption. Among these, adsorption stands out as the most extensively studied, prompting deeper research into different adsorbents, their mechanisms, and how to enhance their efficiency in capturing plastic particles. Although carbon- and metal-based adsorbents dominate current research, biopolymer-based adsorbents are more environmentally friendly due to their biodegradability, low toxicity, and renewable origin. However, a key limitation of adsorption is that it does not achieve complete mineralization of the contaminants. Instead of breaking them down into harmless end products like carbon dioxide and water, adsorption merely transfers the pollutants from one phase to another, which may require further treatment or disposal [4].

Among biological approaches to plastic degradation, microbial degradation has gained significant attention due to its potential for environmentally friendly and complete breakdown of polymers. These methods rely on microorganisms and their enzymes to convert plastics into simpler, non-toxic compounds. A Japanese research team discovered *Ideonella sakaiensis*, a bacterium that can degrade nearly all PET into its monomers, TPA and ethylene glycol (EG), within six weeks at 30 °C using two enzymes: PETase and MHETase [5]. Despite its potential, microbial degradation is often slow and highly dependent on environmental conditions, limiting its scalability and practical application.

Other researchers have investigated various chemical methods for removing PET microplastics. One such study evaluated the effectiveness of the Fenton process combined with coagulation for treating both non-weathered and UV–weathered PET MPs [6]. The use of response surface methodology revealed that increasing both PET and Fe^2+^ concentrations improved removal efficiency in non-weathered PET. These results emphasize the complexity of PET microplastic treatment and the need to consider environmental weathering when designing effective remediation strategies. Meanwhile, a study from Korea University [7] found that ozonation did not degrade PET but caused surface changes, such as cracks and the formation of oxygenated groups, which may enhance adsorption but raise concerns about long-term environmental effects. Other chemical methods, such as hydrolysis, alcoholysis, aminolysis, and pyrolysis, have also been investigated for PET degradation. Recent advances include the use of ionic liquids, eutectic solvents, and metal–organic frameworks as catalysts, which improve depolymerization efficiency and reduce reaction times [8]. However, these methods often require high energy input and may generate secondary pollutants.

Consequently, there is an urgent and critical need for efficient, economically viable, and sustainable degradation technologies to effectively mitigate PET pollution and safeguard water resources. Advanced Oxidation Processes (AOPs) have emerged as promising alternatives for the degradation of recalcitrant organic compounds that are not affected by conventional treatments [9].

AOPs operate by generating highly reactive oxygen species (ROS), primarily hydroxyl radicals (·OH), which possess a high oxidation potential E^0^ = +2.80 V vs. SHE and exhibit non-selective reactivity, enabling the degradation of a wide range of organic pollutants into less harmful intermediates or complete mineralization [9,10].

Recent studies have also demonstrated the potential of persulfate-based AOPs for PET degradation, where singlet oxygen plays a key role in oxidizing PET hydrolysates into valuable products such as formate [11]. These findings highlight the versatility of AOPs not only for pollutant removal but also for resource recovery. Other studies on AOPs highlight the use of peracetic acid [12], ozone [13], and photocatalysis [14] to degrade persistent pollutants like pharmaceuticals. Although PET is not the main focus, these methods could be adapted for its treatment with appropriate pre-treatment, presenting an underexplored research opportunity.

Among the diverse array of AOPs, photochemical processes are particularly attractive due to their ability to induce degradation through direct light absorption or by promoting the formation of reactive species [9]. UV radiation, particularly UV-C, has shown promise in achieving over 90% mineralization of emerging contaminants under environmentally friendly conditions [15]. However, PET remains resistant to UV degradation due to its stable aromatic structure [16]. Among UV types, UV-C is most effective for advanced oxidation processes, while UV-A and UV-B are more commonly used in medical and cosmetic applications [17].

This research specifically explores the application of excimer lamp technology for the photochemical degradation of PET. Excimer lamps, such as KrCl and XeBr, offer distinct advantages over conventional UV sources. They are mercury-free, addressing environmental concerns, and can emit monochromatic UV radiation at specific, highly energetic wavelengths. Specifically, KrCl lamps emit at 222 nm, while XeBr lamps emit at 283 nm [18]. These precise wavelengths are highly effective for initiating photochemical reactions, facilitating the generation of reactive species and accelerating pollutant degradation [19].

A comprehensive study compared the log-reduction doses required for microbial inactivation across three UV spectral ranges: 200–215 nm, 216–225 nm, and 226–235 nm. Results showed that 222 nm radiation (KrCl) was significantly more effective than traditional low-pressure mercury lamps (254 nm), requiring lower doses to achieve the same level of microbial reduction [20].

There is a notable lack of studies addressing the direct degradation of PET through excimer lamp technology, as well as limited characterization of the resulting oxidation intermediates and their potential toxicity. Additionally, the development of hybrid or sequential treatment systems that integrate physical or chemical pre-treatments with AOPs remains insufficiently explored, and little attention has been given to the environmental fate of PET fragments following treatment. Our research group has studied the removal of different compounds (phenols and dyes) with XeBr, KrCl, and Cl_2_ barrier discharge excilamps [21,22,23]. For example, congo red attained total removal in shorter times with the KrCl excilamp for all the molar ratio H_2_O_2_/congo red assayed. This behavior can be explained by the fact that the absorption band for congo red is much higher in the maximum wavelength of emission of the KrCl excilamp (222 nm) than for the wavelength of the XeBr excilamp.

**Figure 1:**
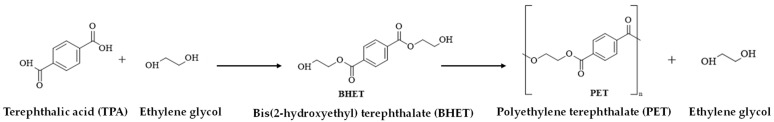
PET synthesis process.

Terephthalic acid (TPA) and bis(2-hydroxyethyl) terephthalate (BHET) are known to be formed as significant hydrolysis or oxidation products during the PET degradation processes since they are the starting monomers in the PET synthesis process as shown in Figure 1. Therefore, studying its degradation behavior provides valuable insights into the broader mechanisms of PET breakdown. This study rigorously investigates, as a first step, the effects of varying the H_2_O_2_/monomer mass ratio, the initial monomer concentration, and the reaction volume on the degradation efficiency of BHET and TPA using both KrCl and XeBr excimer lamps.

## 2. Results

### 2.1. Effect of H_2_O_2_/Monomer Mass Ratio

**Figure 2:**
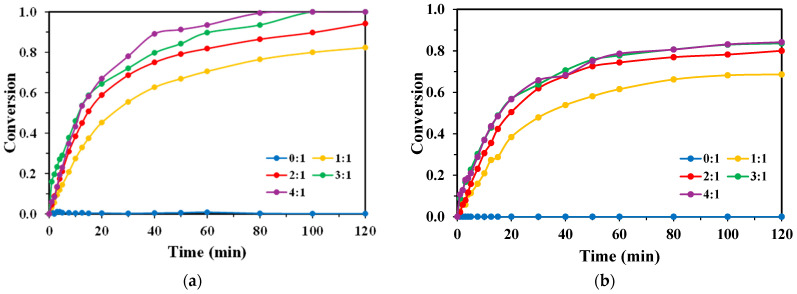
Conversion rates of TPA obtained for each lamp with varying H_2_O_2_:TPA mass ratios, [TPA]_0_ = 100 mg/L, V_R_ = 250 mL, for the (**a**) KrCl lamp; and (**b**) XeBr lamp.

The influence of the H_2_O_2_:TPA mass ratio on degradation efficiency revealed the critical role of hydrogen peroxide as a source of hydroxyl radicals (Figure 2a,b). Direct photolysis (0:1 ratio) in the absence of H_2_O_2_ resulted in negligible TPA removal with both lamps, confirming that UV irradiation alone is insufficient for effective degradation. Specifically, after 2 h of treatment, the TPA removal percentage was only 0.14% with the KrCl lamp, while it was 0% with the XeBr lamp.

For the KrCl (222 nm) system (Figure 2a), degradation efficiency increased significantly with the addition of H_2_O_2_. An optimal performance was observed at a 3:1 mass ratio, which achieved near-complete TPA conversion (>99%) within 100 min. A further increase to a 4:1 ratio did not yield a significant improvement, suggesting that the reaction rate was no longer limited by the oxidant concentration. In contrast, the XeBr (283 nm) lamp (Figure 2b) demonstrated markedly lower efficacy across all ratios. Its highest conversion of approximately 80% was achieved after 120 min at both the 3:1 and 4:1 ratios, highlighting the superior performance of the 222 nm wavelength for this process.

**Figure 3:**
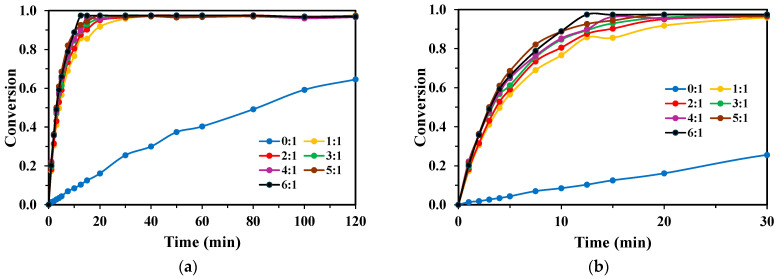
Conversion rates of BHET with different H_2_O_2_:BHET mass ratios, [BHET]_0_ = 100 mg/L, V_R_ = 250 mL, for the KrCl lamp: (**a**) 120 min of reaction; and (**b**) first 30 min of the reaction.

**Figure 4:**
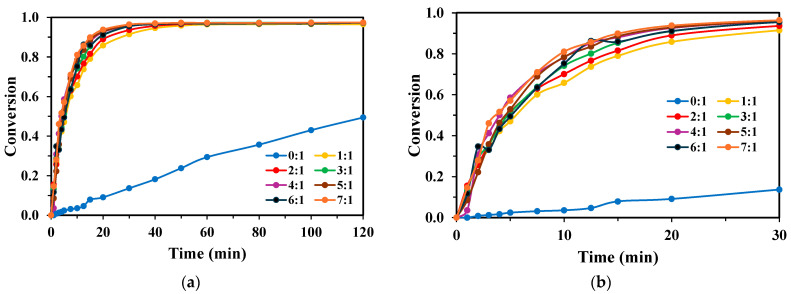
Conversion rates of BHET with different H_2_O_2_:BHET mass ratios, [BHET]_0_ = 100 mg/L, V_R_ = 250 mL, for the XeBr lamp: (**a**) 120 min of reaction; and (**b**) first 30 min of the reaction.

Similar to TPA, the degradation of BHET was highly dependent on the presence of H_2_O_2_ (Figure 3a and Figure 4a). The optimal mass ratio was found to be different for each lamp, reflecting their distinct photochemical efficiencies. For the KrCl lamp (Figure 3a,b), the optimal H_2_O_2_:BHET ratio was determined to be 5:1, achieving complete degradation in approximately 60 min. For the XeBr lamp (Figure 4a,b), the optimal ratio was 4:1, though its overall performance remained inferior to the KrCl system, requiring higher times to approach complete conversion. In both cases, the KrCl lamp initiated degradation more rapidly and achieved completion in a shorter timeframe.

### 2.2. Effect of Initial Monomer Concentration

**Figure 5:**
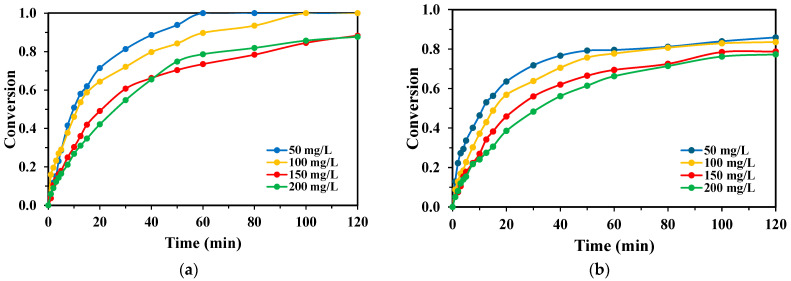
Conversion rates of TPA obtained for each lamp with varying initial TPA concentrations, H_2_O_2_:TPA mass ratio 3:1, V_R_ = 250 mL, for the (**a**) KrCl lamp; and (**b**) XeBr lamp.

The effect of the initial TPA concentration was investigated at the optimal H_2_O_2_:TPA ratio of 3:1 (Figure 5). A distinct inverse relationship was observed between the initial TPA concentration and the degradation rate for both lamp systems. With the KrCl lamp (Figure 5a), degradation was fastest at the lowest concentration, achieving complete conversion at 50 mg L^−1^ in under 60 min. As the initial concentration increased to 150 and 200 mg L^−1^, degradation slowed, reaching final conversions of only 88% and 87%, respectively, within the 120 min experimental period. A similar trend was observed for the XeBr lamp (Figure 5b), though with overall lower degradation rates.

**Figure 6:**
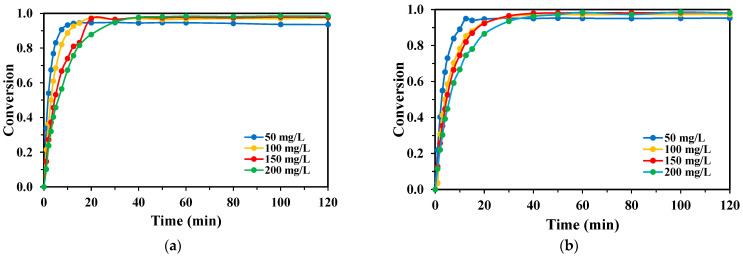
Conversion rates of BHET obtained for each lamp with varying initial BHET concentrations, V_R_ = 250 mL: (**a**) H_2_O_2_:BHET mass ratio 5:1 for the KrCl lamp; and (**b**) H_2_O_2_:BHET mass ratio 4:1 for the XeBr lamp.

The degradation of BHET followed the same inverse relationship with the initial concentration as observed for TPA (Figure 6). Experiments were conducted at the previously determined optimal ratios (5:1 for KrCl and 4:1 for XeBr). For both lamps, the degradation rate was highest at the lowest initial concentration (50 mg L^−1^) and decreased progressively as the concentration was raised to 200 mg L^−1^. The KrCl lamp showed slightly higher efficacy than the XeBr lamp at all tested concentrations.

### 2.3. Effect of Reaction Volume

**Figure 7:**
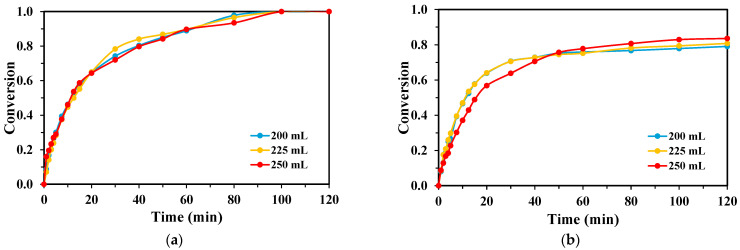
Conversion rates of TPA obtained for each lamp with varying reaction volumes, H_2_O_2_:TPA mass ratio 3:1, [TPA]_0_ = 100 mg/L for the (**a**) KrCl lamp; and (**b**) XeBr lamp.

Variations in the reaction volume between 200, 225, and 250 mL showed only a minimal impact on TPA degradation kinetics (Figure 7). For the KrCl system, complete TPA conversion was achieved across all tested volumes. The XeBr system also showed minor differences between volumes, consistently yielding final conversions below 85%.

**Figure 8:**
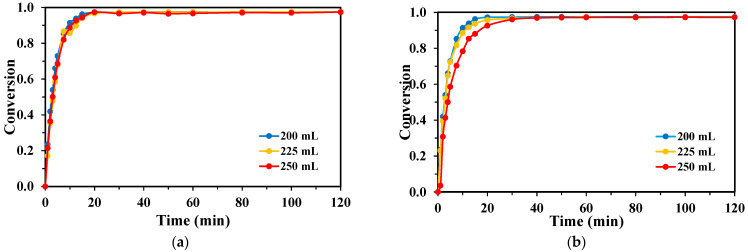
Conversion rates of BHET obtained for each lamp with varying reaction volumes, [BHET]_0_ = 100 mg/L: (**a**) H_2_O_2_:BHET mass ratio 5:1 for the KrCl lamp; and (**b**) H_2_O_2_:BHET mass ratio 4:1 for the XeBr lamp.

The effect of reaction volume on BHET degradation was also found to be minimal, mirroring the results obtained for TPA (Figure 8). The KrCl lamp consistently provided rapid and complete BHET removal regardless of the volume (200, 225, or 250 mL), while the XeBr lamp showed slightly lower performance that was not significantly influenced by volume changes within the tested range.

### 2.4. Chemical Oxygen Demand

**Figure 9:**
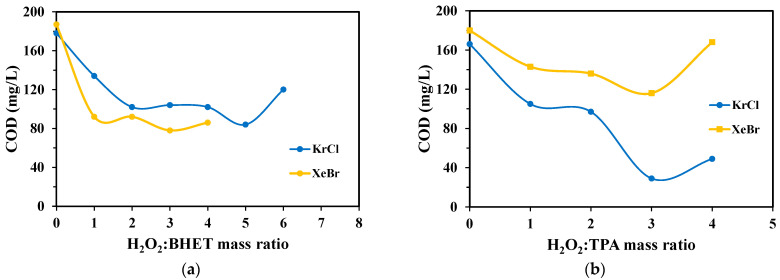
Chemical oxygen demand after irradiation of samples with different H_2_O_2_/monomer mass ratios of (**a**) TPA and (**b**) BHET.

Chemical oxygen demand (COD) measurements were conducted to assess the extent of mineralization for both monomers under optimal H_2_O_2_/monomer ratios. For an initial TPA concentration of 100 mg L^−1^, the theoretical COD is 144.5 mg L^−1^. After 120 min of treatment with the KrCl lamp at a 3:1 ratio, the COD was reduced to 29 mg L^−1^ (Figure 9a), corresponding to an 80% reduction and indicating significant mineralization. In contrast, the XeBr lamp achieved only a minor COD reduction, confirming its lower efficiency.

Different results were obtained for BHET, whose theoretical COD is 157.3 mg L^−1^. The KrCl lamp at a 5:1 ratio resulted in a 50% decrease in COD, whereas the XeBr lamp achieved practically the same COD residual value at a 4:1. Higher mass ratios of 5:1 and 6:1 were tested for the XeBr lamp, but the results showed excessively high out-of-range values.

## 3. Discussion

### 3.1. Experimental Series

#### 3.1.1. Superior Efficacy of the KrCl Excimer Lamp

The most remarkable finding across all experiments is the significantly superior performance of the KrCl (222 nm) excimer lamp compared to the XeBr (283 nm) lamp for the degradation of both TPA and BHET. This observation is consistent with our previous findings on the degradation of other organic pollutants [16,17,18]. The enhanced efficacy is primarily attributed to the photochemical properties of the oxidant. The molar absorption coefficient of H_2_O_2_ is substantially higher at 222 nm than at 283 nm. Consequently, the KrCl lamp achieves much more efficient photolysis of H_2_O_2_, leading to a higher quantum yield for the generation of highly reactive hydroxyl radicals (·OH), the primary species responsible for pollutant degradation. On the other hand, the absorption maxima of terephthalic acid and bis(2-hydroxyethyl) terephthalate (BHET) were found to be approximately 200 nm and 246 nm, respectively. Both are closer to the emission band of KrCl, particularly the maximum absorption wavelength of TPA, which also explains why the degradation of this compound is much better with the KrCl lamp. These results agree with previous studies of the authors using the same excimer lamps to degrade different organic pollutants [21,22,23], with the performance of KrCl being better in all cases.

This higher rate of ·OH generation allows for faster and more complete degradation of the monomers, even though the radiant power of the XeBr lamp (17.12 mW cm^−2^) was greater than that of the KrCl lamp (2.47 mW cm^−2^). This demonstrates that the specific wavelength and its alignment with the oxidant’s absorption spectrum are more critical than the total energy output for the efficiency of this AOP.

#### 3.1.2. Influence of H_2_O_2_/Monomer Ratio and Oxidant Scavenging

The results clearly establish that the UV/H_2_O_2_ process is far more effective than direct photolysis. This agrees with previous studies [16] that concluded that PET and PET–derived compounds are more resistant to direct photolysis than other plastics and, therefore, require the presence of an oxidant agent. However, the existence of an optimal H_2_O_2_/monomer mass ratio highlights a crucial trade-off. Below this optimum, the reaction is limited by the availability of ·OH radicals. Above it, excess H_2_O_2_ acts as a scavenger of hydroxyl radicals, forming the less reactive hydroperoxyl radical (HO_2_·) and thus reducing overall degradation efficiency.

H_2_O_2_ + ·OH → HO_2_· + H_2_O (1)

HO_2_· + ·OH → H_2_O + O_2_ (2)

**Figure 10:**
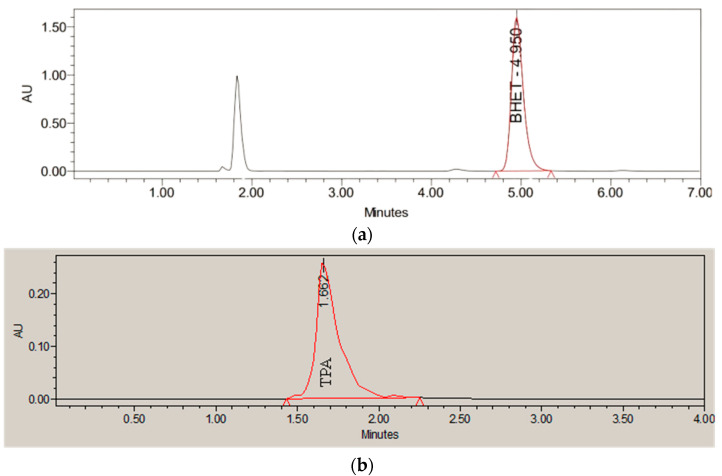
Chromatograms for KrCl excilamp: (**a**) BHET and (**b**) TPA.

This scavenging effect explains why increasing the H_2_O_2_/monomer mass ratio beyond a certain point does not improve the process. The optimal ratio for BHET (5:1 with the KrCl lamp and 4:1 with the XeBr lamp) was notably higher than for TPA (3:1). This is consistent with the molecular structure: BHET (C_12_H_14_O_6_) is a larger molecule than TPA (C_8_H_6_O_4_) and, therefore, has a higher stoichiometric demand for complete oxidation (3.07 for TPA and 3.35 for BHET). Additionally, due to its larger size, photodegradation of BHET with the KrCl lamp leads to the formation of intermediate products that have not been identified (Figure 10). These intermediates also consume hydrogen peroxide during their degradation, which contributes to an increase in the optimal hydrogen peroxide-to-monomer ratio beyond the theoretical value.

#### 3.1.3. Effect of Initial Monomer Concentration and Inner Filter Effect

The observed inverse relationship between initial monomer concentration and degradation rate can be explained by two concurrent phenomena. Firstly, at a constant rate of ·OH radical generation, a higher concentration of the target monomer results in increased competition for these limited radicals, slowing the overall conversion rate.

Secondly, the inner filter effect becomes significant at higher concentrations. Both TPA and BHET absorb UV radiation in the range of the excimer lamps. As their concentration increases, they absorb a greater fraction of the incident photons, effectively shielding H_2_O_2_ molecules from the light. This reduces the rate of photolysis and subsequent ·OH generation, further inhibiting the degradation process.

### 3.2. Chemical Oxygen Demand Analysis

The chemical oxygen demand analysis provides a more comprehensive assessment of treatment efficacy than monomer conversion alone as it quantifies the total oxygen required to oxidize all organic species in the solution.

A key observation for both TPA and BHET was that the final COD after direct photolysis slightly exceeded the theoretical value predicted based on their stoichiometry. This can be due to experimental error. This once again demonstrates that the process without peroxide is not effective.

The substantial COD reduction using an optimal mass ratio confirms that excimer lamp-driven AOP leads to significant mineralization, not only the transformation of the parent compounds.

However, an increase in COD at high H_2_O_2_ concentrations is attributed to two factors. It is partly due to the hydroxyl radical scavenging effect discussed previously, but more significantly, it reflects an analytical interference where unreacted residual H_2_O_2_ is itself oxidized in the COD test, causing a positive error [24].

### 3.3. Fluence-Based Analysis of Degradation Efficiency

To directly compare the energy efficiency of the two excimer lamp systems, monomer conversion was analyzed as a function of fluence (J·cm^−2^) for the optimal mass ratio assays corresponding to each compound and lamp. Fluence not only represents the cumulative UV energy dose delivered to the solution surface but also serves as a direct proxy for the energy cost of the treatment.

**Figure 11:**
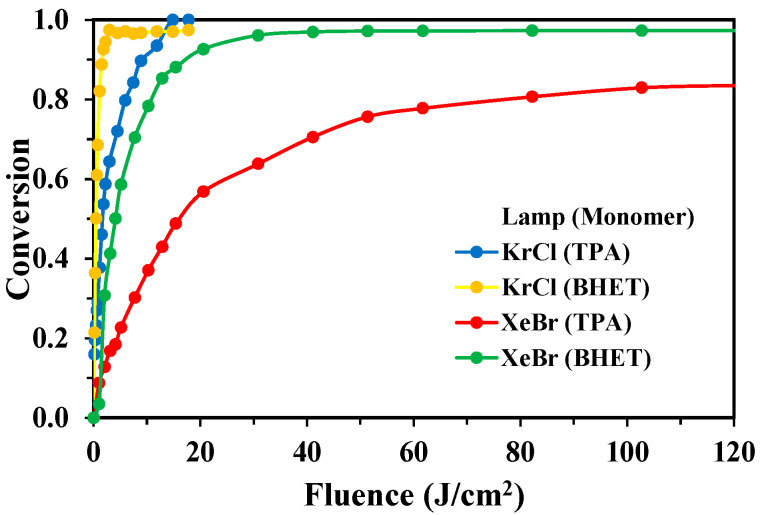
Conversion of TPA and BHET as a function of fluence (UV dose) for the KrCl (222 nm) lamp and the XeBr (283 nm) lamp for the mass ratio H_2_O_2_/monomer optimal assays: (5:1) for BHET with the KrCl lamp, (4:1) for BHET with the XeBr lamp, and (3:1) for TPA with both lamps.

The analysis, presented in Figure 11, reveals the difference in energy efficiency between the two lamps. The KrCl (222 nm; 2.47 mW cm^−2^) lamp achieved complete degradation of TPA at a low cumulative fluence of just 14.82 J cm^−2^. The degradation of BHET was even more rapid, reaching a significant conversion at a fluence of only 2.96 J cm^−2^.

In contrast, the XeBr (283 nm; 17.12 mW cm^−2^) lamp proved far more energy-intensive. Despite delivering a much higher total fluence of up to 123.26 J cm^−2^, the degradation of TPA plateaus at approximately 84%. Moreover, achieving a comparable conversion (>92%) of BHET required a fluence of over 20 J cm^−2^—nearly ten times that of the KrCl lamp. Similar results were observed by the authors in previous studies using KrCl and XeBr lamps as well with dyes as target compounds [19]. This immense difference in energy cost highlights the economic and practical viability of the KrCl–based AOP for PET waste treatment.

## 4. Materials and Methods

### 4.1. Materials

Terephthalic acid (TPA, 98% purity) and bis(2-hydroxyethyl) terephthalate (BHET, 85% purity) were supplied by Sigma-Aldrich (St. Louis, MO, USA) and used as model PET–derived pollutants. Hydrogen peroxide (H_2_O_2_, 33% *w*/*v*), the oxidizing agent, was purchased from PanReac AppliChem (Barcelona, Spain).

A 0.1 M phosphate buffer solution (pH 7.0) was used as the solvent for all experiments to maintain stable pH conditions. It was prepared by mixing appropriate volumes of 0.1 M sodium phosphate dibasic (Na_2_HPO_4_, Sigma-Aldrich) and 0.1 M sodium phosphate monobasic (NaH_2_PO_4_, PROBUS, S.A., Badalona, Spain) solutions. The concentration of the H_2_O_2_ stock solution was periodically verified by titration with potassium permanganate (KMnO_4_, PanReac). Acetonitrile (HPLC grade), ultrapure water, and sulfuric acid (H_2_SO_4_, 98%) were used for preparation of the HPLC mobile phase.

### 4.2. Experimental Setup

Photochemical degradation experiments were carried out in an open, batch configuration. Each reaction was conducted in a 250 mL borosilicate glass beaker placed on a magnetic stirrer (JP Selecta “Agimatic-S”, Abrera, Spain) operating at 700 rpm to ensure complete homogeneity of the solution.

Irradiation was provided by two distinct, mercury-free excimer lamps supplied by the High Current Electronics Institute (Tomsk, Russia):

- A Krypton Chloride (KrCl) excimer lamp with a peak monochromatic emission at 222 nm and an average radiation intensity of 2.47 mW cm^−2^ at the solution surface.
- A Xenon Bromide (XeBr) excimer lamp with a peak monochromatic emission at 283 nm and an average radiation intensity of 17.12 mW cm^−2^ at the solution surface.

**Figure 12:**
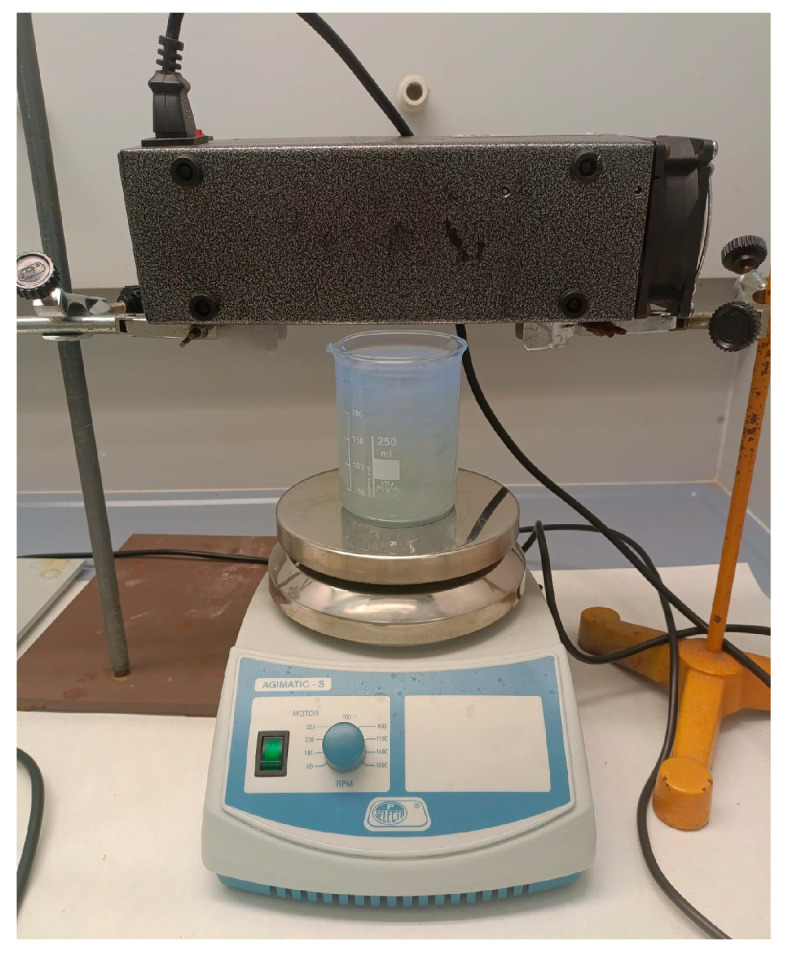
Experimental setup. Borosilicate glass beaker placed on a magnetic stirrer with an excimer lamp positioned 3 cm above the surface of the reaction solution.

For all experiments, the lamp was positioned horizontally 3 cm above the surface of the reaction solution as depicted in Figure 12. The setup allowed for two parallel experiments to be run simultaneously, one for each lamp type.

### 4.3. Experimental Design

The degradation of both compounds, TPA and BHET, was investigated by performing three series of experiments to evaluate the influence of key operational parameters. For each experimental run, aliquots (500 µL) were withdrawn at predetermined time intervals and immediately prepared for analysis. Specifically, sampling was performed every minute for the first 5 min, then at increasing intervals (ranging from 2.5 to 20 min) up to 120 min.

#### 4.3.1. Series 1: Effect of H_2_O_2_/Monomer Mass Ratio

To determine the optimal oxidant dose, experiments were conducted at a constant initial monomer concentration of 100 mg L^−1^ and a fixed reaction volume of 250 mL. The H_2_O_2_:BHET ratios tested ranged from 0:1 to 6:1, while the H_2_O_2_:TPA ratios tested ranged from 0:1 to 4:1 to identify the optimal degradation conditions for each lamp.

#### 4.3.2. Series 2: Effect of Initial Monomer Concentration

The effect of the initial pollutant load was studied by varying the monomer concentration while keeping the reaction volume constant at 250 mL. The H_2_O_2_/monomer mass ratio was fixed based on the optimal conditions determined in Series 1. For BHET, the optimal mass ratios were 5:1 (KrCl lamp) and 4:1 (XeBr lamp) and initial BHET concentrations of 50, 100, 150, and 200 mg L^−1^ were evaluated. For TPA, the H_2_O_2_:TPA mass ratio was fixed at 3:1 with the same initial TPA concentrations being tested.

#### 4.3.3. Series 3: Effect of Reaction Volume

To assess the impact of the solution volume on degradation efficiency, experiments were conducted using volumes of 200, 225, and 250 mL. The initial monomer concentration was held constant at 100 mg L^−1^, and the H_2_O_2_/monomer was fixed at the optimal value for each respective system as in the previous series.

### 4.4. Analytical Methods

#### 4.4.1. Monomer Quantification

The concentrations of TPA and BHET were determined using a Waters High-Performance Liquid Chromatography (HPLC) (Alliance iS HPLC System Software Version 2.0, Waters Corporation, Milford, MA, United States) system equipped with a Waters 600 controller, a Waters 717 plus autosampler, and a Waters 2996 Photodiode Array (PDA) detector. Data acquisition and processing were performed using Waters Empower 2 software (version 2.0). Chromatographic separation was achieved under isocratic conditions on a C_18_ column.

The mobile phase consisted of a mixture of 60% ultrapure water, 20% acetonitrile, and 20% of a 10 mM H_2_SO_4_ aqueous solution. The flow rate was maintained at 1.0 mL min^−1^, and the injection volume was 20 µL. Quantification was performed by integrating the peak area at the wavelength of maximum absorbance for each compound and comparing it against a calibration curve generated from external standards.

#### 4.4.2. Chemical Oxygen Demand Quantification

The extent of mineralization of organic pollutants was evaluated by measuring the Chemical Oxygen Demand. Measurements were performed on selected samples, typically those from the H_2_O_2_/Monomer Mass Ratio series (Series 1), at the conclusion of the 120 min reaction period.

COD was determined using a Hanna Instruments HI 83,099 multiparameter photometer (Woonsocket, RI, United States) and a Hanna Instruments HI 839,800 COD reactor (Woonsocket, RI, United States). The analysis followed the standard dichromate colorimetric method using low-range (LR) commercial reagent vials (HI94754A-0, Hanna Instruments (Woonsocket, RI, United States). Prior to analysis, samples were appropriately diluted (1:2 and 1:4) with ultrapure water to ensure the final COD value fell within the instrument’s calibrated range.

## 5. Conclusions

This study highlights the superior performance of the KrCl excimer lamp (222 nm) over the XeBr lamp (283 nm) in the degradation and mineralization of TPA and BHET via UV/H_2_O_2_ advanced oxidation processes. The presence of H_2_O_2_ was essential for effective degradation, with optimal H_2_O_2_/monomer mass ratios of 3:1 for TPA and 4:1 and 5:1 for BHET with XeBr and KrCl, respectively. Exceeding these ratios led to reduced efficiency due to hydroxyl radical scavenging and analytical interference from residual H_2_O_2_ in COD measurements. Energy efficiency analysis based on fluence confirmed the KrCl lamp’s advantage, achieving complete degradation at significantly lower energy doses compared to the XeBr lamp. Despite its lower radiant power, the KrCl system enabled faster and more complete degradation, attributed to better spectral overlap with H_2_O_2_ absorption and enhanced ·OH radical generation. Overall, the results demonstrate that wavelength selection and oxidant optimization are critical for maximizing AOP efficiency. The KrCl–based system offers a more energy-efficient and effective approach for the treatment of PET–derived pollutants. Although further studies are required to identify intermediate compounds formed along the photodegradation process, the observed results underscore the potential of this technology for the treatment of water contaminated with PET–derived pollutants. Given its efficiency and reliability, further research is warranted to explore its scalability, long-term operational stability, and applicability in real-world wastewater treatment scenarios.

## Data Availability

The data presented in this study are available upon request to the corresponding author.
